# Coated Microneedle System for Delivery of Clotrimazole in Deep-Skin Mycoses

**DOI:** 10.3390/gels10040264

**Published:** 2024-04-15

**Authors:** Barbara Jadach, Agata Nowak, Jolanta Długaszewska, Oliwia Kordyl, Irena Budnik, Tomasz Osmałek

**Affiliations:** 1 Division of Industrial Pharmacy, Chair and Department of Pharmaceutical Technology, Poznan University of Medical Sciences, 3 Rokietnicka, 60-806 Poznan, Poland; 2 Chair and Department of Pharmaceutical Technology, Poznan University of Medical Sciences, 3 Rokietnicka, 60-806 Poznan, Polandtosmalek@ump.edu.pl (T.O.); 3 Department of Genetics and Pharmaceutical Microbiology, Poznan University of Medical Sciences, 3 Rokietnicka, 60-806 Poznan, Poland; jdlugasz@ump.edu.pl; 4 Division of 3D Printing, Chair and Department of Pharmaceutical Technology, Poznan University of Medical Sciences, 3 Rokietnicka, 60-806 Poznan, Poland; okordyl@ump.edu.pl (O.K.); irena.budnik@gmail.com (I.B.)

**Keywords:** microneedles, hydrogel, clotrimazole, antifungal activity, 3D printing

## Abstract

Mycoses of the skin are infectious diseases caused by fungal microorganisms that are generally treated with topical agents. However, such therapy is often ineffective and has to be supported by oral use of active substances, which, in turn, can cause many side effects. A good alternative for the treatment of deep-skin mycoses seems to be microneedles (MNs). The aim of this research was to fabricate and evaluate the properties of innovative MNs coated with a hydrogel as potential carriers for clotrimazole (CLO) in the treatment of deep fungal skin infections. A 3D printing technique using a photo-curable resin was employed to produce MNs, which were coated with hydrogels using a dip-coating method. Hydrogels were prepared with carbopol EZ-3 Polymer (Lubrizol) in addition to glycerol and triisopropanolamine. Clotrimazole was introduced into the gel as the solution in ethanol or was suspended. In the first step of the investigation, a texture analysis of hydrogels was prepared with a texture analyzer, and the drug release studies were conducted with the use of automatic Franz diffusion cells. Next, the release profiles of CLO for coated MNs were checked. The last part of the investigation was the evaluation of the antifungal activity of the prepared systems, and the inhibition of the growth of *Candida albicans* was checked with the diffusion and suspended-plate methods. The texture profile analysis (TPA) for the tested hydrogels showed that the addition of ethanol significantly affects the following studied parameters: hardness, adhesiveness and gumminess, causing a decrease in their values. On the other hand, for the gels with suspended CLO, better spreadability was seen compared to gels with dissolved CLO. The presence of the active substance did not significantly affect the values of the tested parameters. In the dissolution study, the results showed that higher amounts of CLO were released for MNs coated with a hydrogel containing dissolved CLO. Also, microbiological tests proved its efficacy against fungal cultures. Qualitative tests carried out using the diffusion method showed that circular zones of inhibition of fungal growth on the plate were obtained, confirming the hypothesis of effectiveness. The suspension-plate technique confirmed the inhibitory effect of applied CLO on the growth of *Candida albicans*. From the analysis of the data, the MNs coated with CLO dissolved in hydrogel showed better antifungal activity. All received results seem to be helpful in developing further studies for MNs as carriers of antifungal substances.

## 1. Introduction

**Figure 1:**
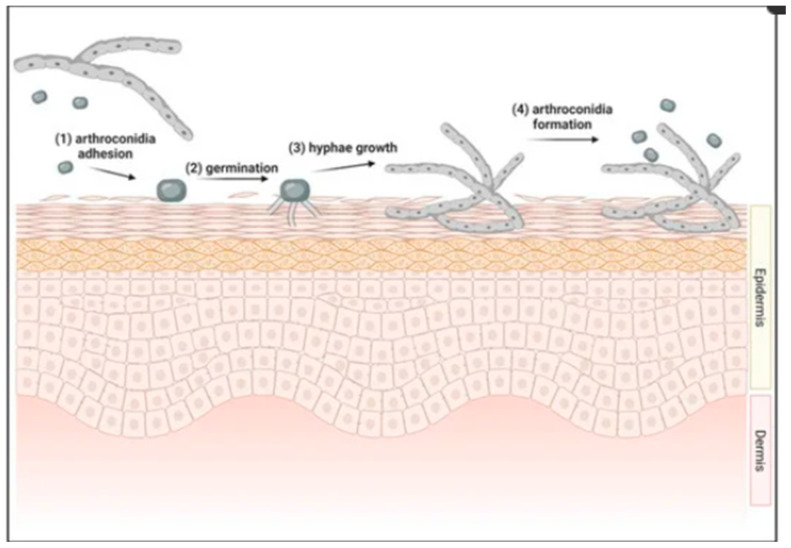
Initiation of dermatophyte infection in skin. (**1**) Arthroconidia from environment or other infected host’s contact with new host’s skin. Adhesion to skin occurs between 2 and 6 h after contact. (**2**) Arthroconidia begins to germinate in top layer of the epidermis, forming germ tubes. (**3**) Hyphae continue to grow within epidermis. (**4**) Within 7 days of infection, arthroconidia are formed, allowing for cycle to repeat (reprinted from [5]).

Mycoses of the skin are infectious diseases caused by fungal microorganisms. It is estimated that up to 20–25% of the world’s population struggles with fungal skin and nail problems. The skin provides ideal conditions for the growth of pathogens [1,2,3]. The most common pathogenic fungi include dermatophytes, yeasts and molds [1,4]. Figure 1 presents the initial stages of the dermatophyte infection in the skin [5].

About 40 species of dermatophytes have been identified, and the three main ones are Trichophyton, Microsporum and Epidermophyton [1], called keratinophilic, which means they possess the ability to decompose keratin in order to obtain a nutritional source, causing infection of the skin, hair, nails and mucous membranes [6]. Antifungal substances play an essential role in the treatment of mycoses. Superficial skin infections are generally treated with topical agents. In the case of larger areas and during immunosuppression or ineffectiveness of the drug used, the infection is combated with oral agents like terbinafine, itraconazole, posaconazole, fosravuconazole, voriconazole and oteseconazole [7,8]. Active pharmaceutical ingredients (APIs) from the imidazole derivatives group are commonly used in local treatment, such as clotrimazole, ketoconazole, econazole, miconazole, etc. An alternative treatment method is the use of terbinafine in a 1% cream or solution for application on the skin [7,9]. In addition to dermal forms, an oral form can be used to increase the effectiveness of the therapy, followed by long-lasting remission of dermatophytosis [10]. Itraconazole is also administered by the same route and is active against a wide range of fungal microorganisms [11,12]. Also, morpholine and allylamine, as well as benzylamino and thiocarbamic acids, are effective. In local antifungal nail therapy, the drug is delivered through the nail plate, which is a safe route of administration and does not cause significant side effects, though it is characterized by limited penetration into deeper areas [9,13]. This leads to frequent relapses of the disease in up to one-fourth of patients [14]. It has been found that of all the external forms, nail polishes provide the best results. Ciclopirox is highly effective against fungi of the genera Candida and Aspergillus [15]. When at least 50% of the nail surface is infected, oral treatment should be administered, including fluconazole, itraconazole, ketoconazole, griseofulvin and terbinafine [9,14]. A global problem of therapy is increasing multidrug resistance, leading to longer hospitalization, reduced effectiveness and, consequently, increasing mortality [16].

The way drugs are delivered to the body is one of the main factors responsible for the effectiveness of antifungal therapy. The most common routes of administration are local and oral [8,10]. Systemic treatment is highly effective but is burdened with serious side effects and numerous interactions with other preparations taken by the patient. Success is also associated with a long treatment period. Formulations applied topically to the skin do not enter the gastrointestinal tract, which avoids undesirable drug metabolism in the liver. But the effectiveness of the therapy may be limited due to the naturally low permeability of the stratum corneum (s.c.), which consequently does not allow for achieving a therapeutic dose [17,18,19]. In the case of mycoses located in the deeper layers of the skin, they are not fully effective; therefore, alternative methods of delivering the compounds are being sought, which will increase skin penetration and improve the effectiveness of treatment [20]. One of the promising ways to solve the above problems seems to be microneedle systems (MNs) [21,22,23]. They effectively bypass the s.c., allowing for more effective local action. MNs are an approach combining the features of local and invasive (injection) therapy [24,25] and also create micro-holes in s.c., improving its permeability [24,26], which allows for free movement of therapeutic macromolecules to the deeper layers of the skin. Due to their small size, microneedles do not reach nerve endings, which makes the application virtually painless [27]. This approach seems attractive to patients, eliminating the emotional aspect. Additionally, the therapy does not require trained medical staff and is available to every patient. The development of MN technology includes new methods of producing systems as well as an increasingly larger group of transferred medicinal substances [24,26,28,29].

**Figure 2:**
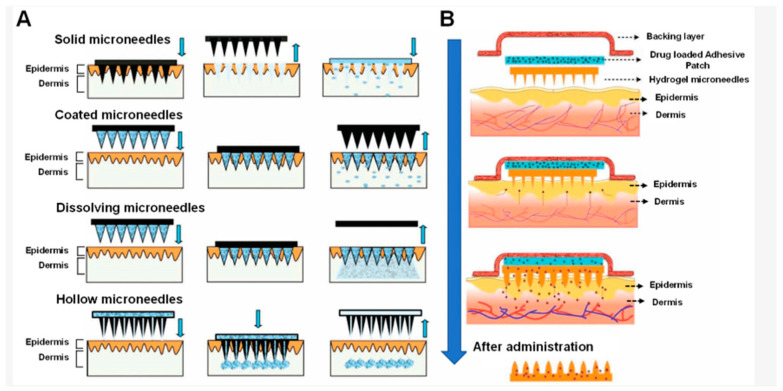
Schematic representation of methods of traditional (**A**) and hydrogel (**B**) microneedle-mediated drug delivery to the skin (arrows point to the order of operations; reprinted from [33]).

In addition to transdermal transport, microneedles have been used in the delivery of drugs to the eye membrane, as well as the mucous membranes of the mouth, nails, vagina and gastrointestinal tract. MNs guarantee accurate and repeatable results, with minimal inter-individual variability in bioavailability [24,25,30]. Also, it is possible to precisely deliver API to the selected site while reducing the dosing frequency [27,31]. Microneedle systems most often have the form of a patch—a matrix with needles (Figure 2) with dimensions of 25–1000 μm [32]. MNs should have adequate strength, ductility and hardness, and also should penetrate the upper layer of the skin—s.c.—without touching the nerves or blood vessels so patients do not feel intense pain during application [27,31].

It is worth mentioning that pain sensations depend on the length of the microneedles, while width, thickness and angle do not have a significant impact [28]. External factors, such as skin physiology and surrounding conditions, are important for proper MN function. Too low humidity delays the release of the drug, while the presence of sweat will prevent the patch from properly adhering to the skin. Raising the skin temperature causes blood vessels to dilate and in this way increases the penetration of API [31]. Despite many advantages, there are some limitations to using MNs, mainly in the form of allergic reactions on the skin and irritations. Moreover, due to the small thickness of the needles, there is a risk of damage and breakage of the tips that will remain in the skin [32,34]. This problem can be solved by selecting the appropriate material for production, which is the leading subject of research during their optimization [24]. Choosing the right composition allows for obtaining a prolonged release of the API under selected conditions [27,32]. There is also a certain risk of polymers being released after the needles are inserted into the skin, but the use of biodegradable polymers combats this drawback and allows for safe use [28].

The aim of this study was to obtain and evaluate the properties of innovative microneedle systems based on 3D-printed matrices as potential carriers for clotrimazole (CLO) in the treatment of deep fungal skin infections [35,36]. The planned research included an attempt to obtain MNs using 3D printing and coating with hydrogels. CLO release for the obtained microneedle systems was investigated with Franz diffusion cells. Finally, efficacy was assessed with microbiological cultures. Additionally, the coating hydrogels were analyzed in terms of texturometric parameters.

**Figure 3:**
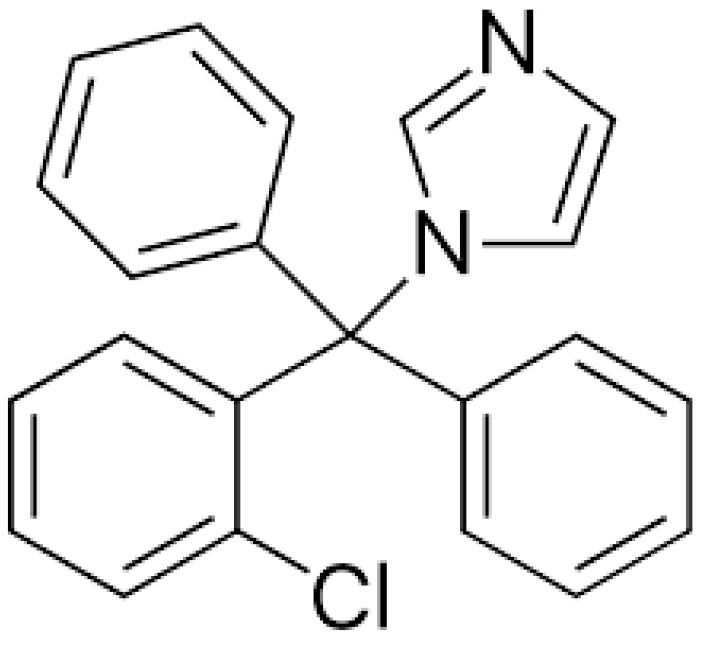
Structure of clotrimazole.

Clotrimazole (Figure 3) is a crystalline, odorless powder with a color ranging from white to pale yellow; it is slightly soluble in water and it dissolves well in ethanol [37,38]. Although it is lipophilic in nature, it has poor absorption when applied to the skin [39]. CLO is available mainly in the form of products for topical use, primarily in the form of creams, solutions and balms; however, after local administration, it has poor bioavailability (ranging from 0.5 to 10%), and the highest concentration remains in the epidermis, especially in the s.c. Due to systemic toxicity and its limited absorption, it is not available in oral form; this means that new carriers for CLO are increasingly being sought to increase drug delivery to the site of infection [39,40].

## 2. Results and Discussion

**Figure 4:**
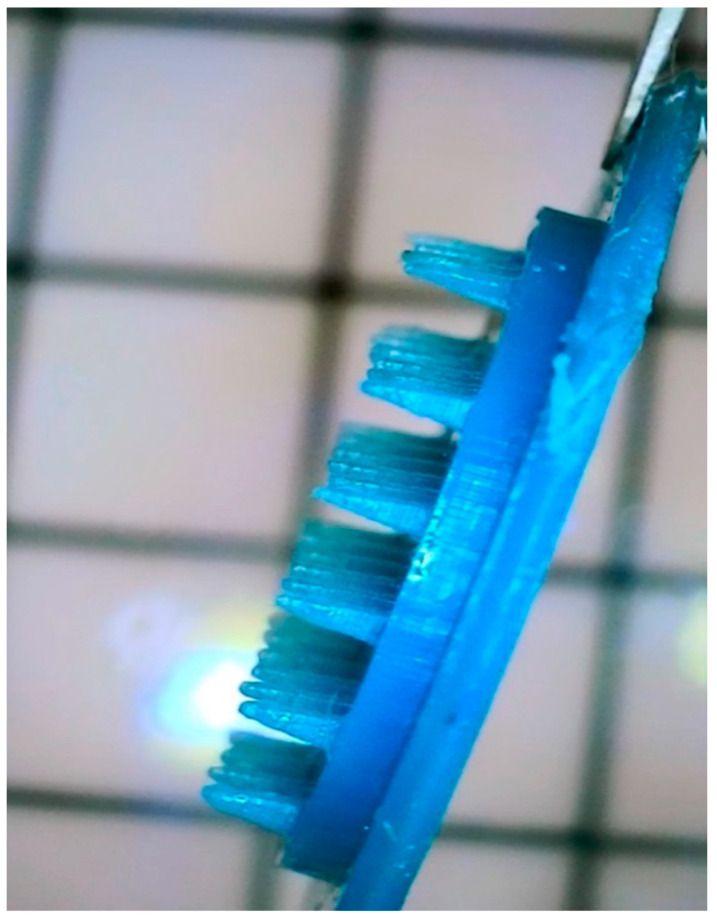
Three-dimensional-printed microneedles coated with the hydrogel.

Microneedles are interesting formulations and are not so popular in the treatment of skin disorders. This study was based on the idea of using MNs as the delivery system into deeper layers of the skin. MNs were successfully printed with 3D printing using a polymer—Phrozen Aqua-Blue photocurable resin. After that, MNs were coated with the hydrogel by the dip-coating method [41], and the received system is presented in Figure 4.

For further tests, two microneedle systems were prepared, coated with ethanolic hydrogel in which CLO was dissolved (MN-KLE), and coated with hydrogel with suspended CLO (MN-KLW).

### 2.1. Texture Analysis of Hydrogels

**Figure 5:**
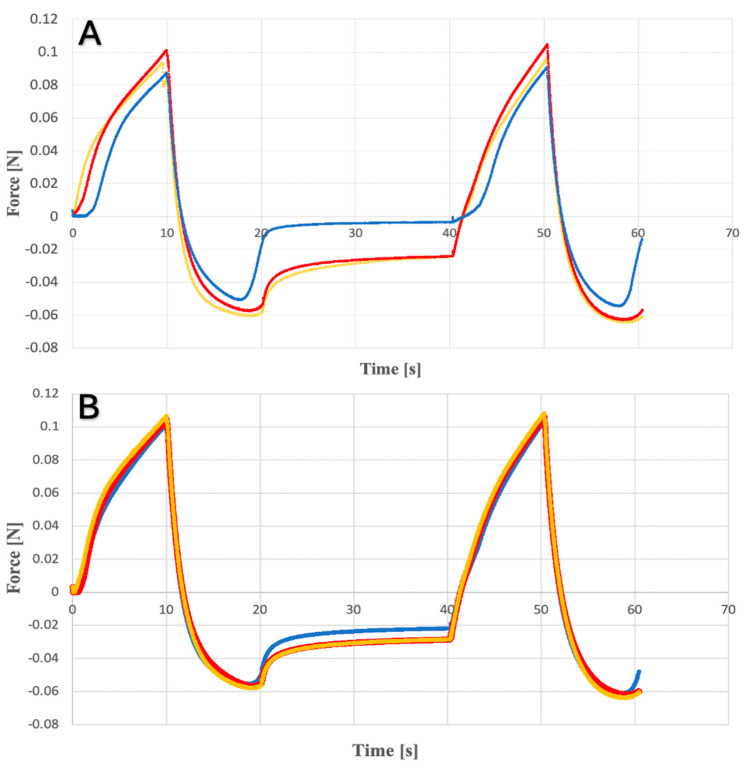
Texture profile analysis for ethanol hydrogels (**A**) with clotrimazole; (**B**) placebo.

**Figure 6:**
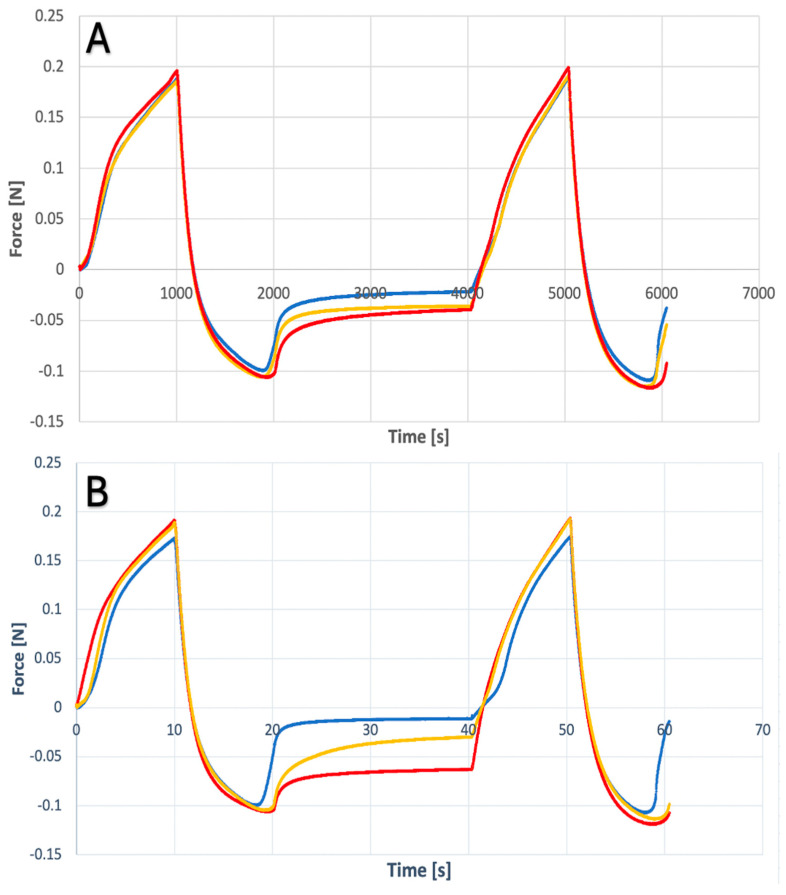
Texture profile analysis for (**A**) suspension hydrogels with clotrimazole; (**B**) placebo.

The results obtained during the study of the prepared hydrogels are presented in Figure 5 and Figure 6 as the relationship between force (N) and time. In Table 1, the basic texturometric parameters are presented.

Based on the received results, it can be observed that the addition of ethanol to the formulation resulted in a decrease in hardness, adhesiveness and gumminess values compared to suspension gels. This is probably related to the formation of hydrogen bonds by alcohol molecules. It probably makes weaker connections inside the polymer and leads to damage to its structure. All tested systems showed a similar cohesiveness value. However, when comparing the parameter values of hydrogels containing CLO compared to placebo hydrogels, no significant differences were found.

### 2.2. Spreadability Analysis of Hydrogels

**Figure 7:**
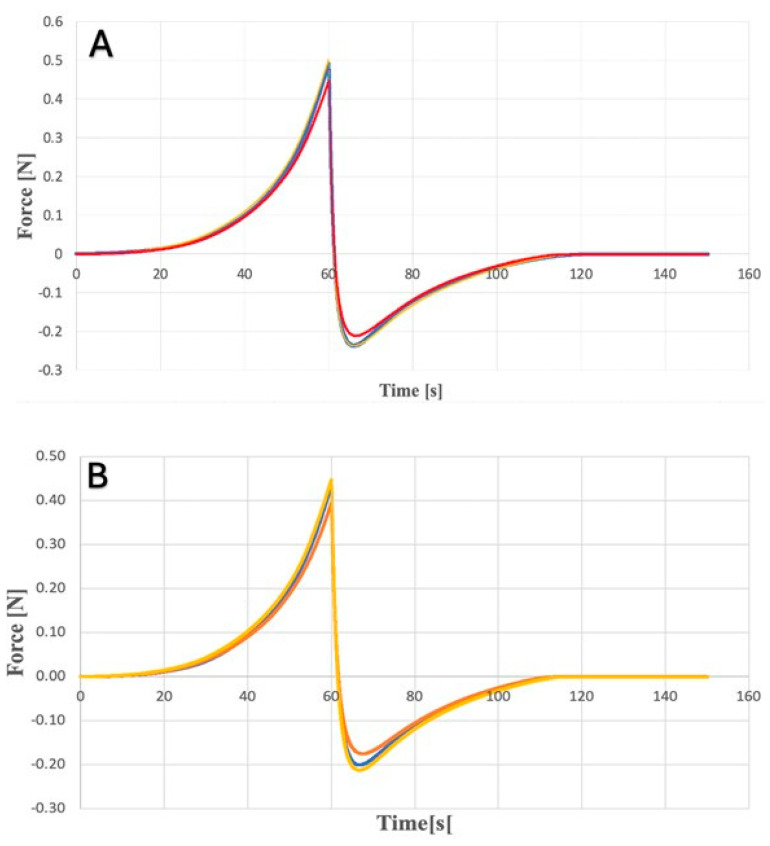
Spreadability profile for ethanol gel (**A**) with clotrimazole; (**B**) placebo.

**Figure 8:**
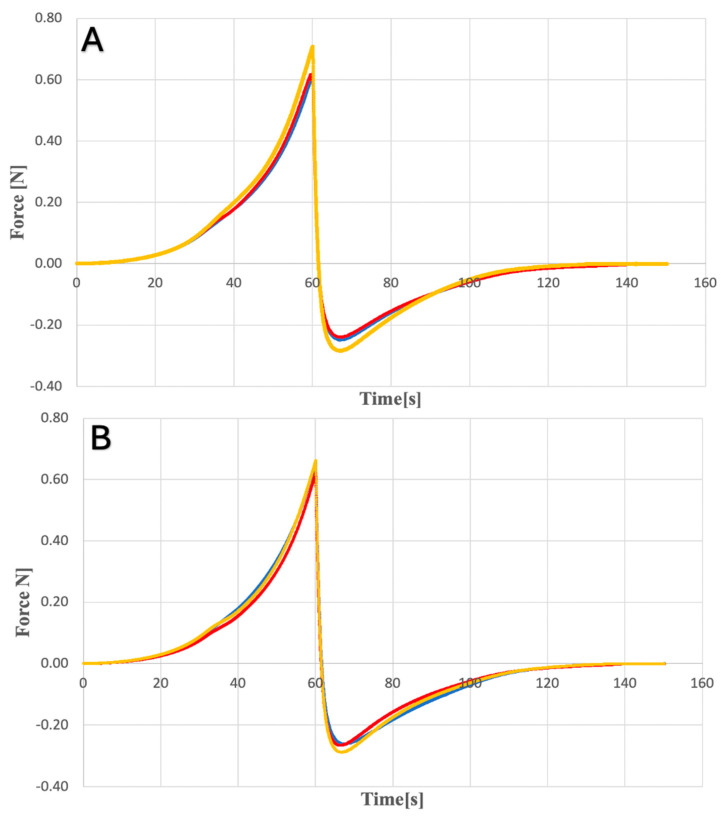
Spreadability profile for suspension gel (**A**) with clotrimazole; (**B**) placebo.

This type of analysis allowed for the assessment of parameters such as strength adhesion, adhesiveness, firmness and spreadability. Analyzing the results, a peak can be distinguished: the maximum of which is firmness, and the area under the peak is spreadability. The negative peak and the area under it are adhesion force and adhesiveness. The obtained values are summarized in Table 2 and presented in Figure 7 and Figure 8 as spreadability profiles.

The analysis of the obtained results indicates that suspension hydrogels have better spreadability. This is due to the lack of ethanol in the system. Glycerin hydrogen bonds dominate, which reduces the stiffness of polymer chains and facilitates the spreading of the gel [42]. Similarly to the texture study, no significant differences were found when the parameter values of hydrogels containing CLO were compared to placebo hydrogels.

### 2.3. Dissolution Study

**Figure 9:**
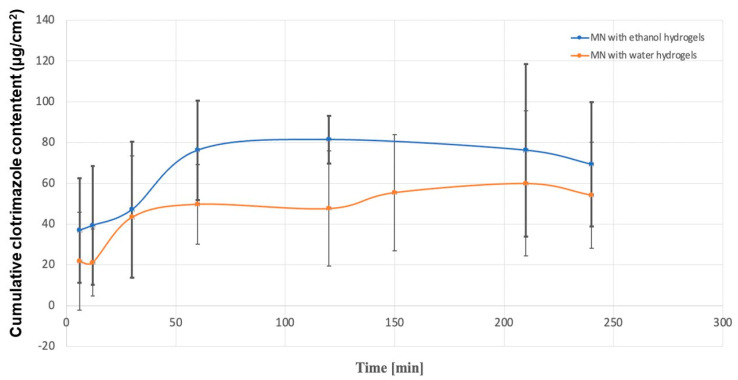
Comparison of CLO release for MN-KLE (blue line) and MN-KLW (red line).

Dissolution tests were performed for each hydrogel (KLE, KLW) and microneedle system (MN-KLE, MN-KLW), and the cumulative CLO content per unit area was compared. For all tested systems, the obtained release profiles were non-linear. The results show (Figure 9) the dependence of the accumulated content of clotrimazole in the acceptor fluid for the remaining time.

**Figure 10:**
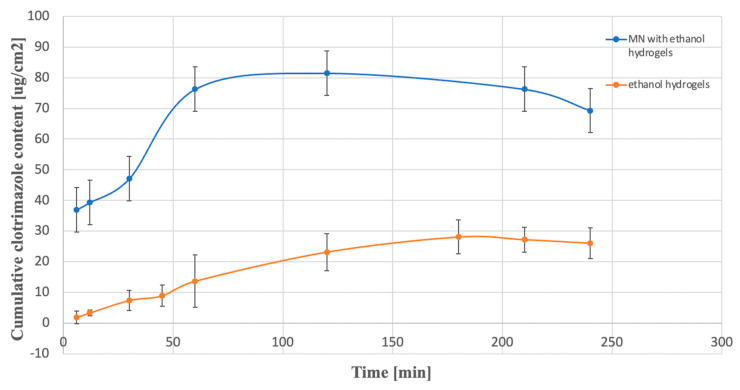
Comparison of the release profiles of CLO for MN-KLE (blue line) and for KLE (red line).

Figure 9 shows that MN-KLE released a higher amount of CLO than MN-KLW. This is probably due to the good solubility of clotrimazole in ethanol and weak solubility in water. After 12 min of testing, releases of approximately 39 µg/cm^2^ and 21 µg/cm^2^ were observed for MN-KLE and MN-KLW, respectively. The initial release rate of CLO was two times higher for MN-KLE than MN-KLW. A sharp increase in the release of CLO was observed, especially between 12 and 30 min for MN-KLW and between 30 and 60 min for MN-KLE. After 30 min, the cumulative content of CLO for both tested MNs showed a similar value of approximately 45 µg/cm^2^. For MN-KLE, approximately 76 µg/cm^2^ of CLO was released after 60 min during the study; an equilibrium state was established and the amount of the API in the medium did not increase at subsequent time points. In the case of MN-KLW, a smaller amount of CLO was released after 60 min than for the MN-KLE—i.e., approximately 50 µg/cm^2^—and a further increase was observed at subsequent time points in the amount of clotrimazole in the medium. Further, after 150 min and 210 min, approx. 55 µg/cm^2^ and 60 µg/cm^2^ were released, respectively. The reason for the observed changes is probably the slower washout of CLO from the suspension gel resulting from the gradual dissolution of the solid crystals, while in the ethanol gel, clotrimazole, which was already being dissolved, was more easily transferred to the medium in the acceptor chamber. When testing the dissolution of CLO from hydrogels, a lower cumulative content of clotrimazole in the acceptor fluid was observed compared to coated MNs. A comparison of the release profiles for MN-KLE and KLE is shown in Figure 10.

In the case of the gel, no sudden increase in the accumulated CLO content was observed. The obtained values were significantly lower than the amounts of CLO released for MN-KLE. After 60 min of the test, this value was approx. 14 µg/cm^2^ for the dissolution of the gel and 76 µg/cm^2^ for MN-KLE. A similar relationship was observed when examining the releases for KLW and MN-KLW. Already in the initial stage of the study, a two-times higher value was shown for MN-KLW (approx. 43 µg/cm^2^) than for the gel (approx. 17 µg/cm^2^), while after 120 min, the value of the released API was 48 µg/cm^2^ for microneedles and 34 µg/cm^2^ for the gel. It can therefore be concluded that applying the gel to the microneedles significantly improves the release profile of the active substance. The obtained differences are caused by direct contact of the MN with the tested fluid, unlike gels, which are separated by a semi-permeable membrane. Different results of this study could be achieved if the membrane was replaced with living tissue.

### 2.4. Analysis of Antifungal Activity

#### 2.4.1. Diffusion Method

**Figure 11:**
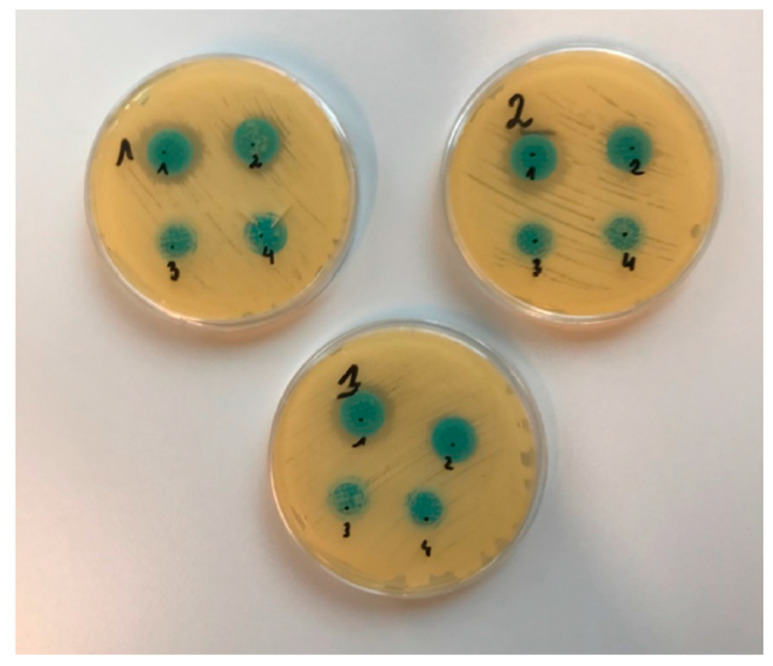
Visible zones of growth inhibition in diffusion test (1. MN-KLE; 2. MN-KLW; 3. MN-PLE; 4. MN-PLW) (*n* = 3).

After 24 h of incubation, growth-inhibition zones were observed for microneedles containing the active substance (MN-KLE, MN-KLW), which were not observed for placebo microneedles (Figure 11). The inhibition growth zones of *Candida albicans* colonies were measured with a ruler, and the results are presented in Table 3.

The observation of the plates and analysis of the data collected in Table 3 showed that MN-KLE inhibits the growth of *Candida albicans* better than MN-KLW, suggesting better antifungal properties. When considering the above result, two possible causes were taken into account. Firstly, the effect obtained could be due to the good solubility of CLO in ethanol, which resulted in better diffusion of the drug in the medium, guaranteeing its higher concentration. The next subject of consideration was the antifungal activity of ethanol itself; it was expected that its fungicidal effect would significantly affect the result of the experiment. However, this statement is questionable because, while observing the plate, no zone of growth inhibition was observed where the microneedle with the placebo ethanol gel was placed (MN-PLE), and therefore the hypothesis was rejected.

#### 2.4.2. Suspension-Plate Method

**Figure 12:**
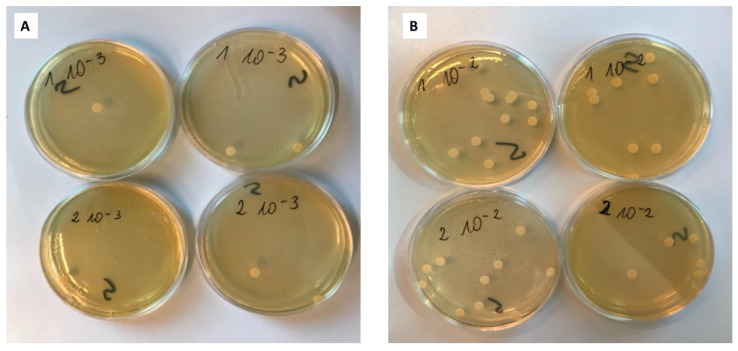
(**A**) Plates with surface inoculation at time t_0_ after 48 h of incubation in 10^−3^ dilution (1. MN-KLE; 2. MN-KLW); (**B**) plates with surface inoculation at time t_0_ after 48 h of incubation at dilution 10^−2^ (1. MN-KLE; 2. MN-KLW).

After 48 h of incubation, the number of *Candida albicans* colonies on each plate was counted (Figure 12) and the number of overall colonies was calculated. The results of the study are presented in Table 4 and Table 5.

Analyzing the above tables, it can be seen that the calculated number of colonies at time t_0_ (immediately after adding the fungi) for the control plate is similar to the number of colonies in the tested plate. However, the differences are not significant in the interpretation of the results. However, the culture performed at time t_12_ deviates significantly from the initial state. A lower number of fungal colonies was obtained for plates with CLO compared to the control plate. In turn, when analyzing the control plates at times t_0_ and t_12_, a clear increase in the number of colonies should be noted, much higher (approx. four logs) than in the case of sample k1 (approx. 1.5 log). This proves the effective inhibition of fungal growth and demonstrates a positive test result for the effectiveness of microneedle systems against microbiological cultures.

## 3. Conclusions

Analyzing the results obtained during this study, the following conclusions can be stated. The addition of the active substance does not affect the texture profiles of the prepared gels. The presence of ethanol in Carbopol EZ-3 hydrogels causes a significant decrease in the hardness, adhesiveness and gumminess. The developed MNs with CLO are effective against fungal cultures. Systems coated with ethanol hydrogel have higher drug release rates, followed by a better ability to inhibit fungal growth in comparison to suspension gels. All results obtained appear to be helpful in developing further studies for MNs as carriers of an antifungal substance. Such drug delivery seems to be effective in action and could be helpful for the treatment of fungal disorders located in the deeper layers of the skin, if not as an independent therapy, then as a support for oral treatment.

## 4. Materials and Methods

### 4.1. Materials

Clotrimazol was purchased from Pol-Aura (Poznan, Poland). Carbopol^®^ EZ-3 was purchased from Lubrizol Corp. (Wickliffe, OH, USA). Glycerol was purchased from Fagron (Kracow, Poland). Ethanol 96%, isopropanol and sodium chloride were purchased from Avantor Performance (Gliwice, Poland). Phosphate buffer concentrate was purchased from Chempur (Piekary Śląskie, Poland). Resin was purchased from Phrozen Aqua-Blue (Hsinchu City, Taiwan). Triizopropanolamine was purchased from Sigma-Aldrich (St. Luis, MI, USA). Sabouraud dextrose and yeast culture broth were purchased from Merck KGaA (Darmstadt, Germany).

### 4.2. Preparation of Microneedles

**Figure 13:**
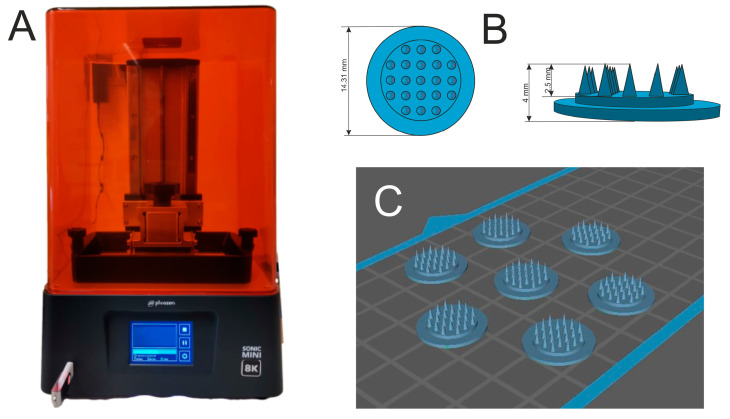
(**A**). Phrozen printer; (**B**) project of microneedles, (**C**) 3D slicer image of printed needles.

The microneedles were obtained by 3D printing using a Phrozen Sonic Mini 8K printer (Figure 13A), which is based on LCD technology.

This printer uses an LED module with linear projection as the light source. The printing resolution is very high and reaches 22 μm. The material used was a—Phrozen Aqua-Blue photocurable resin. The MNs planned for preparation (Figure 13B) consist of 21 cone-shaped needles located on a circular base with a diameter of 14.31 mm. The height of one needle is 2.5 mm, the total height of the microneedles is 4 mm and its radius is 4 mm—base 1 mm. The printed MNs were placed in a container of isopropanol and then cured in the Anycubic Wash&Cure 2.0 curing chamber by placing the MNs on a moving platform that rotates 360° for even curing with UV light.

### 4.3. Preparation of the Hydrogels

Two types of hydrogels were used to coat the microneedles, containing the same dose of clotrimazole suspended in gel (KLW) or dissolved in ethanol and mixed with gel (KLE). Also, the same placebo hydrogels were prepared without active substances (PLW and PLE). The polymer used to prepare the hydrogel was Carbopol EZ-3 Polymer (Lubrizol), while triisopropanolamine was used as a neutralizing agent. To obtain an ethanol-based hydrogel, glycerol and deionized water were weighed in a 100 mL plastic container. Carbapol was added to the surface of this mixture and samples were mixed while covered at a speed of 600 rpm for 25 min. Then, CLO was dissolved in ethanol in the glass beaker and the solution was transferred to the sample of the prepared gel; stirring was continued in the same conditions for 5 min. After mixing, the previously prepared 40% solution of triisopropanolamine in water was added dropwise while gently mixing with a glass rod. Everything was transferred to a glass container (KLE). The placebo hydrogel (PLE) was prepared analogously, replacing 0.5 g of the active substance with 0.5 g of deionized water, added in the first stage of gel preparation. In order to obtain the suspension hydrogel, glycerol and deionized water were weighed in a 100 mL plastic container and Carbopol was added to the surface of the mixture. The samples were mixed while covered at 600 rpm for 30 min. After this time, they were mixed with prepared 40% solution of triisopropanolamine in water and added dropwise while gently mixing with a glass rod. The weighed CLO was mixed in the mortar with a small amount of gel to form “concentrate” and then the rest of hydrogel was mixed until a homogeneous structure of the hydrogel with suspended active substance (KLW) was obtained. Prepared gel was transferred to a glass container. The placebo hydrogel (PLW) was prepared analogously, replacing 0.5 g of the active substance with 0.5 g of deionized water, added in the first stage of gel preparation. The compositions of prepared placebo and active substance hydrogels are presented in Table 6.

### 4.4. Coating of Microneedles

**Figure 14:**
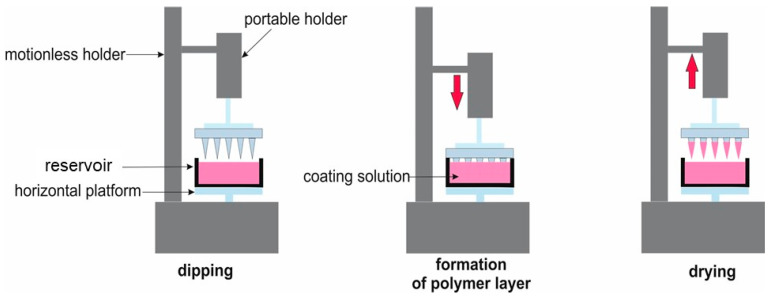
Schematic dip-coating process (reprinted from [43]).

The microneedles were coated with the above-described hydrogels using the dip-coating method, which involves immersing the object in the material and leaving it to evaporate volatile substances at room temperature. The process flow diagram is shown in Figure 14.

Coating was performed using the moving mechanism of the 3D printer by immersing the printed MNs once in the hydrogel tank [43]. The painting was viewed under a microscope (Figure 4).

### 4.5. Characterization of Prepared Hydrogels and Microneedles

#### 4.5.1. Texturometric Profile of Hydrogels

This analysis provides information on the behavior of hydrogels subjected to stress. The test was performed using a Shimadzu AGS texturometer, and a steel, cylindrical probe was used. Hydrogel samples were placed in glass 25 mL beakers in a way that prevents the formation of spaces containing air, and their surfaces were leveled using a metal spatula. The analysis was performed by double compression of the measurement probe moving at a speed of 60 mm/min, immersing to a depth of 10 mm. The resting time between compressions was 20 min. The experiment was repeated three times for three different samples. The obtained results were analyzed using TrapeziumX version 1.5.2 software and graphed as dependence of force on time. Based on the analysis, the following parameters were determined: adhesion, compressibility and hardness. Hardness is defined as the maximum peak at the beginning of compression, adhesiveness is the negative surface area between the peaks, and cohesiveness is a ratio area of the obtained peaks, and gumminess is the product of hardness and cohesiveness. This research was aimed at taking into account the dependency of change parameters depending on the presence of the active substance and the presence of the water and alcohol phases.

#### 4.5.2. Spreadability Analysis

This study assesses the ease of spreading samples by measuring the force required to spread the gel between the upper and lower holders. The test was performed on the same device as the texture analysis, using a cone-shaped attachment (45° angle). Samples were prepared in a similar manner by placing the gel in the lower cap, removing air spaces. The measurement was carried out by squeezing the probe once, dipping to a depth of 10 mm and returning to approximately 5 mm. The probe movement speed was 10 mm/min. The analysis was repeated for three different samples, and the results are presented in the form of a graph. The analysis allowed for the assessment of parameters such as adhesion strength, adhesion, firmness and spreadability.

#### 4.5.3. Dissolution Study of Clotrimazole for Hydrogels

This study of the release of CLO for gel-coated MNs and the hydrogels themselves was carried out using automatic diffusion chambers Phoenix DB-6. The acceptor fluid filling the Franz diffusion chambers was a 20% solution ethanol in phosphate buffer with pH = 7.4. The buffer was prepared by mixing ready-made phosphate buffer concentrate (Chempur) and deionized water in 1:25 ratio. SERVA synthetic membranes (VISKING C/150 dialysis tubing) were used to analyze the release of CLO for hydrogels. Membranes were placed in acceptor liquid for an hour to soak before the examination, then placed in Franz diffusion cells between the donor and acceptor chambers. Three tests were performed for each type of hydrogel (*n* = 3). Firstly, chambers were prepared, refilling with 9.5 mL of acceptor fluid as well as a glass bottle (containing 20 mL of fluid) intended to replenish the collected quantity. Samples of the gel for testing were applied to the chamber donor using syringes. For this purpose, 5 mL of hydrogels were collected using tared Injekt Luer Solo syringes, and then the filled syringes were weighed; approximately 1.5 mL of hydrogel was transferred to Franz chambers and the weight of the syringe was checked again. The mass difference is the amount of preparation placed in the donor chamber. The release process was tested at a temperature of 32 °C and a medium mixing speed of 200 rpm. At specific time points, 200 μL of fluid was collected from each acceptor chamber, transferred to HPLC vials and the missing volume was replaced with fresh release fluid. A validated HPLC method was used to determine the amount of released CLO. The test for the dissolution of CLO for MNs (KLE and KLW) was carried out in a similar way by placing a gel-coated needle in the donor chamber instead of a gel. The MN was in direct contact with the acceptor fluid (without the use of a synthetic membrane). To calculate the value of released CLO, Equation (1) was used:

(1) $$Q=\frac{{Cn\times V+\Sigma }_{i=1}^{n-1}Ci\times S}{A}$$

where *Q*—cumulative content of active substance; $\Sigma_{i=1}^{n-1}$—sum of concentration of active ingredient in *n* − 1 point of probe; *V*—volume of the Franz cell (mL); S—volume of sample (mL); A—surface of the diffusion (cm^2^).

#### 4.5.4. Analysis of Antifungal Activity

The analysis of activity against fungal microorganisms was carried out using two different methods: the diffusion method (qualitative) and the suspension-plate method (quantitative). This study aimed to determine the sensitivity of fungi of the genus *Candida* to CLO delivered by MNs. The analysis began with strain preparation. For this purpose, *Candida albicans* ATTC 10231 was cultured in Sabouraud dextrose broth (Merk KgaA, Darmstad, Germany) for 24 h at 35 ± 1 °C, maintaining aerobic conditions. After this time, the fungal cultures were centrifuged for 15 min (3000 rpm), suspended in a 0.85% sodium chloride solution and then diluted in a suitable medium to the desired concentration.

**The diffusion method** is based on the phenomenon of drug diffusion in the medium. The malt extract agar (Merk KgaA, Darmstad, Germany) plates were inoculated with 100 μL of fungal suspension in a 0.85% sodium chloride solution containing c.a. 1 × 10^8^ CFU/mL (Colony Forming Unit). The suspension was spread with a disposable sterile spatula over the entire surface. In order to determine the sensitivity of the drug, four systems of MNs (MN-KLE, MN-KLW, MN-PLE, MN-PLW) were placed in the marked places, directing the needles towards the substrate. Placebo MNs were created as a controlled trial. To confirm the correctness of the result, three identical plates were prepared. The plates were incubated for 24 h at 35 ± 1 °C. The measure of the sensitivity of microorganisms to an antifungal drug is the diameter of growth inhibition, measured with a ruler (with an accuracy of 1 mm). A circular zone of growth inhibition characterizes a standard test.

**The suspension-plate method** is a combination of two methods: the suspension and agar plate counting methods. It allows for determining the number of microorganisms per unit of volume in a sample. In the initial research stage, the fungal suspension in Sabouraud dextrose broth, containing c.a. 2.0 × 10^5^ CFU/mL, was prepared. For two glass bottles (k1 and k2), MNs containing the active substance were transferred, 2 mL of Sabouraud dextrose broth was added, and the samples were inoculated with 100 μL of the *Candida albicans* suspension. The number of viable cells was determined using the plate count method immediately after sample preparation (t_0_) and after 12 h (t_12_) of incubation at 35 ± 1 °C. For this purpose, the samples were diluted ten-fold (10^−2^ and 10^−3^) in a sodium chloride solution, plated on the Malt extract agar and incubated at 35 ± 1 °C for 48 h. Two samples were performed for each dilution to calculate CFU. The control sample was a culture without the active substance (broth inoculated with the inoculum). At the 48th hour of the incubation, the numbers of fungal colonies on each plate were counted, and the number of CFU for the samples was calculated with Equation (2).

(2) $$N=\frac{\Sigma C}{V(n_{1}+0.1\times n_{2})\times d}$$

where ΣC —sum of colonies on all plates from two successive dilutions; *n*_1_—numbers of plates of first dilution; *n*_2_—numbers of plates from second calculated dilution; d—dilution coefficient; V—volume of inoculation applied to each plate (mL).

#### 4.5.5. HPLC Analysis

The HPLC with UV–Vis spectrophotometric detection and with isocratic elution was used to determine the content of CLO during test detection. This study was performed using a Nexera-i LC-2040C liquid chromatograph (Shimadzu) by setting the following parameters: mobile phase: 55%—acetonitrile solution, 45%—phosphate buffer solution with pH = 9; mobile phase flow rate: 0.75 mL/min; sample (injection) volume—20 μL; column temperature—40 °C; detection at wavelength—210 nm; column: Kinetex pheomenex; Phenyl-Hexyl 100, pores 2.6 μm, dimensions: 100 × 3 mm (Shim-pol). Validation was performed in accordance with ICH guidelines, illustrating the quality of the obtained results measurements. In order to plot the standard curve, measurements were carried out on five concentration levels, performing three repetitions for each concentration. During validation, parameters such as linearity, precision, accuracy and assessment of method specificity were determined.

## Figures and Tables

**Table 1:** Parameters calculated from texture profile analysis obtained for the hydrogels.

Type of Hydrogel	Adhesivness (mJ)		Cohesivness (-)		Hardnes (mN)		Gumminess (mN)	
	Medium	SD	Medium	SD	Medium	SD	Medium	SD
KLE	−0.40	7.09 × 10^−5^	0.90	0.11	93.54	0.003	84.39	0.007
PLE	−0.39	1 × 10^−5^	0.91	0.01	104.33	0.002	94.66	0.002
KLW	−0.68	4.16 × 10^−5^	0.88	0.44	193.19	0.005	169.80	0.006
KLP	−0.66	5.5 × 10^−5^	0.86	0.033	185.72	0.009	159.78	0.010

**Table 2:** Parameters calculated from spreadability analysis for hydrogels.

Type of Hydrogel	Adhesiveness (mJ)		Force of Adhesion (mN)		Firmness (mN)		Spreadability (mJ)	
	Medium	SD	Medium	SD	Medium	SD	Medium	SD
KLE	−0.72	0.0002	−185.43	0.05	403.94	0.09	0.80	16.0 × 10^−5^
PLE	−0.73	0.0001	−196.48	0.02	425.36	0.03	0.85	6.0 × 10^−5^
KLW	−1.20	0.0001	−285.92	0.04	685.64	0.06	1.53	9.7 × 10^−5^
PLW	−1.19	0.0001	−272.12	0.01	646.40	0.02	1.43	7.5 × 10^−5^

**Table 3:** Inhibition growth area in the diffusion method.

Type of Microneedles	Inhibition Growth Area (mm)		
MN-KLE	24	25	20
MN-KLW	19	17	17
MN-PLE	0	0	0
MN-PLW	0	0	0

**Table 4:** Number of colonies counted at time t_0_.

Type of Plate	Number of Counted Colonies		Number of CFU/mL
	Dilution 10^−2^	Dilution 10^−3^	
1	13	1	1.1 × 10^4^
1	8	2	
2	10	2	7.5 × 10^3^
2	5	1	
control	5; 6	1; 0	5.5 × 10^3^

**Table 5:** Number of colonies counted at time t_12_.

Type of Plate	Number of CFU/mL
k1	2.1 × 10^5^
control	7.2 × 10^7^

**Table 6:** Composition of prepared hydrogels.

Ingredient	KLE	PLE	KLW	PLW
	[g]	[g]	[g]	[g]
Carbopol EZ-3	0.5	0.5	0.5	0.5
Ethanol 96%	25.17	25.17	-	-
Glicerol	5.0	5.0	5.0	5.0
Clotrimazol	0.5	0	0.5	0
Triizopropanolamine	0.75	0.75	0.75	0.75
Water	18.08	18.58	43.25	43.75

## Data Availability

The data presented in this study are openly available in article.
